# Tiny Medicine: Nanomaterial-Based Biosensors

**DOI:** 10.3390/s91109275

**Published:** 2009-11-19

**Authors:** Yeo-Heung Yun, Edward Eteshola, Amit Bhattacharya, Zhongyun Dong, Joon-Sub Shim, Laura Conforti, Dogyoon Kim, Mark J. Schulz, Chong H. Ahn, Nelson Watts

**Affiliations:** 1 Nanoworld and Smart Materials and Devices Laboratory, College of Engineering, University of Cincinnati, OH, 45221, USA; E-Mail: force9488@gmail.com; 2 Davis Heart & Lung Research Inst, Biomedical Engineering Dept. The Ohio State University, OH, 43210, USA; E-Mail: Edward.Eteshola@osumc.edu; 3 Environmental Health, College of Medicine, University of Cincinnati, OH, 45267, USA; E-Mail: bhattaat@uc.edu; 4 Internal Medicine, College of Medicine, University of Cincinnati, OH, 45221, USA; E-Mail: dongzu@ucmail.uc.edu (Z.D.); conforl@ucmail.uc.edu (L.C.); 5 BioMEMS Lab, College of Engineering, University of Cincinnati, OH, 45221, USA; E-Mail: kim.2508@osu.edu; 6 College of Dentistry, The Ohio State University, OH, 43210, USA; E-Mail: kim.2508@osu.edu; 7 Nanoworld and Smart Materials and Devices Laboratory, College of Engineering, University of Cincinnati, OH, 45221, USA; E-Mail: Mark.J.Schulz@email.uc.edu; 8 BioMEMS Lab, College of Engineering, University of Cincinnati, OH, 45221, USA; E-Mail: chong.ahn@uc.edu; 9 University of Cincinnati, Bone Health and Osteoporosis Center, College of Medicine, OH, 45221, USA; E-Mail: nelson.watts@uc.edu

**Keywords:** tiny medicine, nanomaterials, point of care

## Abstract

Tiny medicine refers to the development of small easy to use devices that can help in the early diagnosis and treatment of disease. Early diagnosis is the key to successfully treating many diseases. Nanomaterial-based biosensors utilize the unique properties of biological and physical nanomaterials to recognize a target molecule and effect transduction of an electronic signal. In general, the advantages of nanomaterial-based biosensors are fast response, small size, high sensitivity, and portability compared to existing large electrodes and sensors. Systems integration is the core technology that enables tiny medicine. Integration of nanomaterials, microfluidics, automatic samplers, and transduction devices on a single chip provides many advantages for point of care devices such as biosensors. Biosensors are also being used as new analytical tools to study medicine. Thus this paper reviews how nanomaterials can be used to build biosensors and how these biosensors can help now and in the future to detect disease and monitor therapies.

## 1. Introduction

With the advent of nanotechnology, biosensing is entering a new era in the development of advanced sensors that can detect low level concentrations of analytes using a portable device, which was impossible in the past [1-4]. New nano-materials which have high strength, good electrical conductivity, nanoscale size, and that are compatible with biological molecules are ideal for developing biosensors with a low detection limit [1-4]. This paper reviews recent advances in the area of nano-based biomaterials for the development of biosensors in medicine.

**Figure 1.**
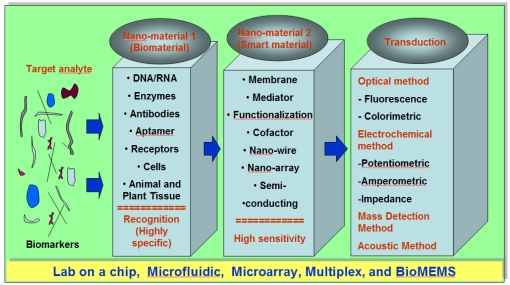
Outline for the development of nanomaterial-based biosensors.

Figure 1 is an outline for the development of nanomaterial-based biosensors. The outline consists of three parts; (1) Nanomaterial 1 (biomaterial), (2) Nanomaterial 2 (smart material), and (3) Transduction. The function of Nanomaterial 1 is basically to recognize a target molecule (biomarker) in solution using the unique selectivity of our biological system. DNA and antibodies were mainstreamed in the past as the recognition layer since they only react with a specific DNA sequence or protein. Now nanobiology with the help of molecular biology and synthetic biochemistry have been developing new artificial recognition elements such as aptamers and a single chain variable fragment which is highly specific to a certain protein, which are collectively designated as Nanomaterial 1. The function of Nanomaterial 2 is to amplify the binding event using a novel property such as a change in the electrical conductivity of a nanowire. Nanowires and nanoparticles that are based on organic or inorganic materials show extraordinary optical, magnetic, mechanical, and electrical properties. Physical modification of these nanomaterials to form membranes, array patterns, and porous structures has been explored for sensor devices. Quartz crystal microbalance (QCM) and micro-cantilever methods of amplification are currently receiving increased attention [5,6]. The third part of sensor development is to transmit the signal generated from Nanomaterial 2 to a final display. Efficient instrument design and signal conditioning will be the primary design drivers in developing a signal transmission system, with the concern of sampling time, amplification, and electromagnetic induction (EMI) shielding.

Small biosensor devices should be integrated or packaged using a proper fabrication technique. Recent advanced micro-electro-mechanical-systems (MEMS) technologies including microelectronics, microfabrication, microarray, photolithography, and micromachining that are applied to the biomedical research area are called BioMEMS. Incorporation of BioMEMS into a microfluidic channel within the sensor and further lab-on-a-chip development will allow more automatic small-volume sampling, pre-separation, multi-protein detection, and waste treatment. The BioMEMS platform provides various functionalities and cost reduction for medical diagnostics. Another advantage of lab-on-a-chip technology is that multiple-protein or multiple-DNA detection can be performed by immobilizing different recognition proteins or DNA on the microarray surface and by multiplexing over the sensor elements. Using multiple sensing elements will provide more quantitative and accurate results. However, integration of a sensor device into a lab-on-a-chip is challenging since it is difficult to obtain repeatable and reliable results. Thus calibration of sensors should be carefully investigated.

When a biosensor is considered for a clinical purpose, sensitivity from a low to high detection limit, linearity range, selectivity which can minimize interference from possible chemicals, the stability of Nanomaterials 1 and 2, reproducibility, and the response time of the sensor should be carefully thought out before designing the sensor. Thus, the sensor data should be clinically meaningful and practical. Based on this biosensor concept, the following sections will provide a very brief review and examples of Nanomaterial 1, Nanomaterial 2, and Transduction through an integrated chip to detect biomarkers in medicine.

## 2. Advances in Sensor Recognition Proteins (Nanomaterial 1)

Biosensors are powerful new analytical tools that combine the exquisite selectivity of biological recognition elements with the processing power of modern micro/opto-electronics and nanostructured materials. Biosensors will have major applications in medicine and molecular diagnostics, drug discovery, environmental monitoring, bio-security, agriculture, food, and in the processing industries. The micro/opto-electronic sensing components are designed to capture electronic (current, potential, capacitance), optical, chemical, and biochemical signals that are generated mainly through molecular interactions when the biorecognition elements specifically interact with the target analyte(s) of interest. There are two major types of biosensors that are based on the type of biorecognition element that is immobilized onto the surface of the physicochemical transducer. The first type is catalytic biosensors which utilize enzymes, cells, tissues/organelles, and microorganisms for the recognition agent. The second type is affinity biosensors which utilize whole antibodies, antibody fragments, nucleic acids/aptamers, receptors, lectins, phages, novel engineered scaffold-derived binding proteins, molecularly imprinted polymers/plastic antibodies and synthetic protein binding agents as the recognition agent [5-16]. The marriage of biology and micro- and nano-fabrication technologies has revolutionized biosensing and led to integration of biological recognition elements into sensing devices that will significantly impact commercially available detection and diagnostic sensing at the genome, proteome, and other levels [17,18].

**Figure 2.**
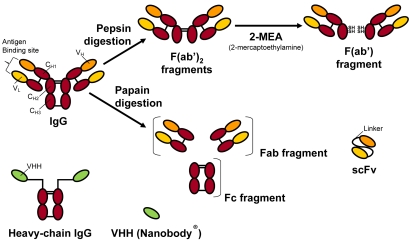
Schematic structure of conventional and heavy-chain IgG and their fragments. Papain digestion of conventional IgG produces Fab fragments and fragmented Fc domains while pepsin digestion yields F(ab')_2_ that can be further reduced to Fab' fragments. VHH fragments are produced by species of the *Camelidae* (e.g., camel, llama, *etc.*) and cartilaginous fish (e.g., sharks).

**Figure 3.**
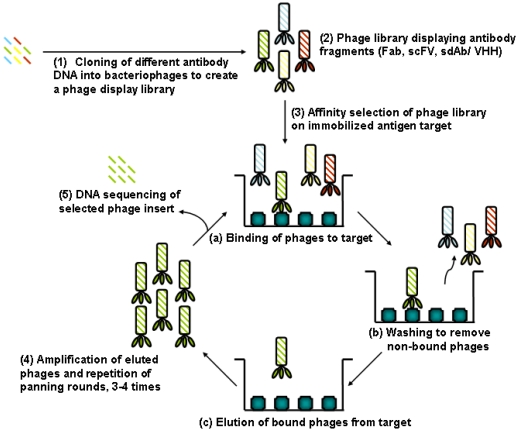
Schematic showing steps involved in affinity selection to isolate target analyte specific antibody (Ab) fragments (Fab, scFv or sdAb/VHH) from an antibody phage display library: (1) DNA encoding millions of different Ab fragments (Fab, scFv or sdAb) is cloned into the genome of filamentous bacteriophage linked to one of the phage coat protein genes; (2) Each DNA variant is packed into a separate phage particle, and the Ab fragment displayed on the phage coat protein; (3) Phage displaying Fab, scFv or sdAb that bind to the target analyte are selected using biopanning cycles of (a) binding, (b) washing, and (c) elution; (4) Eluted phages are reinfected into *E. coli* cells and amplified for further rounds of affinity selection; (5) Clones from the enriched library are characterized for binding properties using appropriate techniques.

Apart from the quality and characteristics of the sensor surface and the interactions at the liquid-solid interface, the properties of the biorecognition elements to be used for analyte binding is the other major factor that strongly affects the performance of bioaffinity sensor systems. Although new and improved sensors will continue to be developed, the more crucial need in any affinity biosensor platform may be the quality of the molecular recognition component (e.g., antibody, aptamer, nucleic acid, receptor, *etc.*) [11,12,17,18]. Hence, improvements in the affinity, specificity, dynamic range of analyte detection, stability and mass production of the molecular affinity recognition components may ultimately dictate the success or failure of detection technologies in both the technical and commercial sensor. The voluminous body of published literature under the title of ‘biosensor’ would prevent presentation of a detailed description of the above mentioned biorecognition elements as well as different transducing elements and the varied devices currently available. Therefore, the brief overview in this section will be restricted to a few selected affinity molecular recognition elements currently shaping the field of emerging biosensor platforms (Figures 2 and 3).

The historical progress of affinity biosensor technology indicates that much effort has been devoted to using naturally occurring biomolecules (e.g., polyclonal and monoclonal antibodies, enzymes, and receptors) that have some inherently desirable binding or enzymatic characteristics to fit a biosensor [20]. Thus, the traditional development of most biosensors has involved the identification of a naturally occurring bio-macromolecule with the required specificity, choosing a suitable signal, and construction of a detector adapted to the properties of the biomolecule in question [20]. While biosensor platforms that were developed following this approach have improved tremendously over the past two decades, the results of adopting naturally occurring biomolecules to fit a biosensor or relying solely upon use of the intrinsic properties of biological molecules in biosensor development has not been as successful as expected in terms of selectivity, sensitivity and stability [21-23]. Thus, it is obvious that while the structure and function of the wide variety of natural biological macromolecules is impressive, fabrication of biomaterials-based devices or systems is inherently limited by the available diversity, cross-reactivity, and stability problems of native proteins used as biosensor recognition elements [24-26].

This realization has led to increasing and concerted efforts by research scientists around the world to embark on the development of a new generation of biosensor recognition elements that are not naturally occurring but ones that have been molecularly engineered and synthesized in the laboratory. Thus, current research trends in biosensor design and fabrication have been shifting from modifying synthetic sensing surfaces towards the engineering (designing and synthesizing) of suitable interfacial recognition nanobiomaterials. Examples of these novel and emerging biorecognition elements include phage display derived and enzyme engineered antibody fragments (Figures 2 and 3), aptamers, novel binding protein scaffolds, synthetic protein binding agents (peptoids), plastic antibodies, and others. These new biorecognition elements are being developed for the molecular or nanoscale modification and functionalization of sensor surfaces and interfaces for the sensing of target analytes of interest [6-18,21-26]. Recent advances in molecular biology and protein engineering techniques, in combination with polymer and bioorganic chemistries, bioconjugation techniques, and surface bio/chemistries [15,27], are allowing the engineering and optimization of biorecognition molecules. There is also the possibility for developing genetically engineered and bioinspired biorecognition nanobiomaterials which contain all the essential functionalities (e.g., size, specificity, affinity, stability, charge characteristics, *etc.*) required for modern bioaffinity sensor design and development.

To this end, techniques of combinatorial biology display technologies and enzymatic engineering are being used to prepare a number of different sized antibody fragments (e.g., single domain [sdAb], single chain antigen binding fragment [scFv], and antigen-binding fragments [Fab]). These fragments are being employed for the systematic modification and functionalization of the sensing channel surface/interface in a biologically modified field effect transistor (BioFET) sensor with potential applications in transplant rejection monitoring and other biomedical areas. The studies are aimed at improving the efficiency of charge transfer (and hence sensitivity of the biosensor device) due to analyte binding to receptor proteins (in this case, the engineered antibody fragments). The receptor itself is also bound to the sensing channel surface.

Several antibody fragment libraries have been successfully biopanned against a chemokine antigen of interest, Table 1 in the subsection below shows results which demonstrate successful biopanning of single chain antibody fragment phage display libraries against the chemokine antigen.

### 2.1. Antibody and Antibody Fragments Based Recognition

With the notable exception of the glucose sensor, the majority of rapid detection systems employ whole antibodies (monoclonal and polyclonal), and increasingly smaller antigen-binding antibody fragments obtained through enzymatic engineering and combinatorial biology display technologies (Figures 2 and 3) for recognition and quantification of target analytes. Antibody recognition elements make use of the high sensitivity and specificity of biomolecular antibody-antigen interactions. There exists a large body of published literature on the subject of generating antibody fragments using enzymatic engineering of whole antibody molecules (Figure 2). Discussed here is the current use of combinatorial biology based library systems (e.g., phage display) for the selection of reduced size antibody fragments with specific affinity to analyte targets of interest. Specific biopanning enrichment processes (Figure 3) are discussed. Examples of such minimal size binders are the antigen-binding fragment (Fab, ∼50 kDa), the single chain antigen binding fragment (scFv, ∼25 kDa), and the single domain antibody fragment (sdAb/VHH). The sdAbs found in camelid and some shark species are unique and are also the smallest known antigen binding antibody fragments (∼12–14 kDa).

Phage display (PD) is a well established example of *in vitro* and *in vivo* biology-based combinatorial display technologies. Phage display allows the isolation of target-specific functional antibody fragments from large libraries containing billions of different antibody fragment sequences. PD has been widely used since the demonstration of the linkage between phenotype and genotype in filamentous bacteriophage [28]. The display of proteins on the surface of phage is accomplished by inserting genes encoding the antibody fragment (or protein of interest) into the genome of the phage via fusion to a viral coat-protein gene. This results in the physical linkage of genotypes and phenotypes of the displayed protein, while keeping their spatial structure and biological activity relatively independent. Large numbers of infectious particles can be propagated conveniently by “amplification” in male Escherichia coli. Thus, large libraries of variant antibody fragments (with complexities >109) presented on phage can be conveniently constructed. As mentioned above, the presented variant antibody fragments frequently are in a configuration that allows them to bind specifically to known or unknown analyte/affinity targets. Iterative affinity selection procedures allow screening of libraries of displayed poly/peptides for library members able to bind affinity reagents of interest. As mentioned above, Table 1 contains results of successful isolation of binders against an analyte of interest from phage display libraries of single chain antibody fragments.

Thus, phage display technology is a powerful biological combinatorial tool for discovering novel antibody fragments that bind to specific or unknown target bioreagents. It has tremendous advantage in its ability to synthesize highly diverse combinatorial libraries biologically, and (depending on the coat protein used as a fusion partner and the choice of the system) to express, on the phage surface, different types of antibody fragments (Fabs, scFvs, sdAbs/VHHs) in their active conformations [29-32]. The affinity, selectivity and stability of antibody fragments affinity isolated against a given target analyte can be further improved or fine tuned by such protein engineering techniques as mutagenesis or recombination [11,12]. In addition to antibody fragments, aptamers and peptide nucleic acids (PNAs) are being actively investigated as recognition elements for sensor applications [33,34]. Aptamers are artificial nucleic acid ligands which can be synthesized against certain targets such as proteins and drugs.

From this brief overview, it is obvious that a great deal of scientific attention has and is currently being given to the area of molecular affinity recognition elements. Achieving the ultimate goal of widespread availability of miniature, sensitive and accurate affinity biosensor platforms will depend on advances on molecular biology, molecular engineering, and polymer and bioorganic chemistries. If recent scientific progress is a fair indicator, the future promises remarkable new developments in molecular affinity recognition elements for use in biosensors with a plethora of applications [6-19].

## 3. Sensing Nanomaterials (Nanomaterial 2)

Once the recognition layer of the biomaterial is decided, this biomaterial should be immobilized on the surface of the smart nanomaterial, which has unique physical properties. Smart nanomaterials should be functionalized to attach specific proteins, which can be accomplished by acid treatment, plasma treatment, and polymer coating. Then, the biomaterial can be covalently bonded to the smart nanomaterial using proper coupling agents such as 1-ethyl-3-(3-dimethylaminopropyl) carbodiimide hydrochloride (EDC), *N*-hydroxysulfosuccinimide (NHS), and *N,N*′-dicyclohexyl carbodiimide (DCC) for activated amidation on the surface of the functionalized material. Each step of the fabrication process such as functionalization and bioconjugation should be well characterized to get quantitative results. Scanning electron microscopy (SEM) and transmission electron microscopy (TEM) with energy dispersive X-ray analysis (EDX), X-ray photoelectron spectroscopy (XPS), Fourier transform infrared spectrometry (FT-IR), Raman spectroscopy, fluorescence imaging, atomic force microscopy (AFM), and scanning tunneling microscopy (STM) are well-known surface sensitive techniques used to characterize the structure, chemical and physical properties of nanomaterials.

Smart nanomaterials for electrochemical sensor use can recognize a binding event and amplify and transfer a signal to the transduction part of the sensor. Smart nanomaterials were explored using carbon nanotubes and semiconducting nanowires. The nano size of an electrode in solution changes its diffusion property to radial diffusion, which eventually increases sensitivity and lowers the detection limit. One electrode that is nano size can conjugate one or a small number of biomolecules, which can detect a low concentration of target molecules. The semiconducting effect of individual nanowires such as ZnO, and Si nanowires was used [35] for the development of highly sensitive biosensors by gating the potential. This nanostructure was aligned as arrays, which increase the detection range and detect multiple proteins simultaneously. Potentiometric, amperometric, and impedimetric techniques are widely used to transduce the change in analyte to an easily interpretable electronic signal.

**Figure 4.**
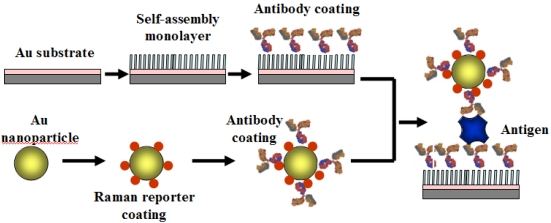
SERS-based immunosensor platform; a gold nanoparticle is coated with Raman-sensitive used for secondary-antibody label (Modified from [33]).

Smart materials for optical sensors were studied using nanomaterials such as quantum dots and gold shells, which are coupled with optical analytical tools. Analytical tools such as Surface Plasmon Resonance (SPR) coupled with optical fibers have improved over the past 20 years [36-46], and can continuously monitor an analyte in real time without requiring a labeling procedure. With the recent progress of opto-electronics, the integration of SPR to lab-on –a chip was successfully done. Further modification with nanomaterials can increase the sensitivity by supporting surface plasmon or localized surface plasmon. For example, Zin *et al.* proved that quantum dot (QD) nanoarrays increased up to 15-fold surface-plasmon-enhanced fluorescence. Surface enhanced Raman scattering (SERS) is an emerging technique since it is based on ultrasensitive, non-isotopic, non-fluorescent detection, with a signal enhanced by a factor of 10^−14^ by electromagnetic nanomaterials such as Au and Ag [38]. Figure 4 shows a SERS-based immunosensor platform. Optical tuneability by changing the dimension of the nanomaterial allows the best sensitivity for not only sensing specific proteins but also for imaging cells and tissues. Especially important is that the photoluminescence properties of cadmium selenide and zinc sulfide quantum dots, and gold (Au) and silver (Ag) nanoparticles, depend on the particle size and shape.

Recent developments in the synthesis of novel nanoparticles has moved lateral flow immunoassay (LFI) to a next generation of advanced detection technique. These prefabricated strips containing dry reagents that are activated by applying a fluidic sample were modified with nanoparticles such as gold, quantum dots, and silicate, which eventually increased sensitivity. Since LFI takes only 20 minutes as a point of care device, one of the well-commercialized applications is in the area of pregnancy test and other applications such as heart attack, and infectious diseases [47-49].

## 4. Device Fabrication and Characterization

With the development of nano- and micro-fabrication technologies, two layers of nanomaterials (biomaterial and smart material) can be embedded into a tiny device, where the sensitivity and linearity range can be tuned by changing active sensing area or geometry. To realize functional devices with these materials, various researchers have patterned nanoscale features and reliably assembled nanomaterials by such techniques as Electron Beam Lithography (EBL), Nano Imprint Lithography (NIL), Dip-pen lithography, Micro Contact Printing (μCP) and various self assembly approaches [50-57]. As an advanced step to achieve a smart system to detect multiple analytes using complex array of functional elements, these nanoscale-sensing materials have been plugged into an analytical lab-on-a-chip platform [58,59]. The lab-on-a-chip incorporating functional nanomaterials can automatically perform biochemistry experimental procedures such as sample preparation, chemical mixing, and detection of target molecules with real time monitoring [60,61]. Additionally, because this miniaturized system precisely processes the laboratory measurement with a small quantity of chemical reagents, lab-on-a-chip technology attains high accuracy and fast processing of biological tests in a cost-efficient manner on a small size chip [62]. Among various applications of this innovative technology, point-of-care testing (POCT) is one of the most promising areas due to its requirement for a multifunctional and miniaturized device that is disposable [63,64].

**Figure 5.**
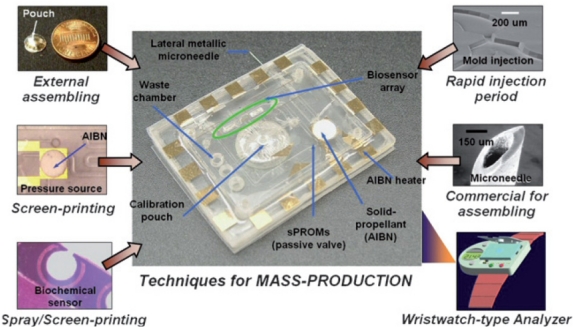
Disposable multifunctional biochip with wristwatch-type analyzer for POC clinical diagnostics (Reprinted with permission from [65]).

In order to fully explore the advantages of LOC devices for POCT applications, the device should be fabricated in a mass-producible manner with low cost materials. A principle technique for the fabrication of these chips relies on photo-definable organic materials and the subsequent processes such as deposition, etching and molding. One of the representative advances in this research field is plastic injection molding developed by Ahn *et al.* [64,65]. Injection molding can be performed with low cost materials, long durability of the mold, and high speed chip production (1 chip/minute). Therefore, this technique is very desirable for mass production of disposable LOC products with multifunctional components. Figure 5 shows a disposable multifunctional biochip with a wristwatch-type analyzer for POC clinical diagnostics.

The major challenge with these small biochips is the accuracy of the sensing performance. Large machines in hospitals use complex optical and magnetic systems to analyze the target samples with high sensitivity and reliability. However, to develop small size sensors and systems, achieving competitive performance with conventional facilities is the major objective of development. For this purpose, nano-scale materials are very promising as a sensing component, because a large surface to volume ratio provides a sensitive response to surrounding media by changing electrical, optical and chemical properties [66-71]. Also, the small size of nanostructured material is very desirable for integration with LOC technology.

**Figure 6.**
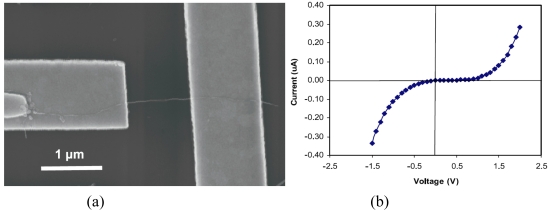
CNT biosensor: (a) SEM image of the assembled SWNT; and (b) I-V curve showing its semiconducting property (Reprinted from [71]).

In order to assemble the nano materials in LOC biochips, various methods have been tried such as dielectrophoretic force (DEP) [72], chemical templating [73], and dip-pen lithography [70]. Replacing optical lithography with electron beam lithography enables patterning a nanosize sensing area. Dip-pen nanolithography using nanoink allows the patterning of different proteins on the sensing area. Besides detection of a specific protein by coating antibody or aptamer on the sensor active element, coating a specific protein can be done by culturing live cells to study signaling networks. Shim *et al.* recently reported the fluidic self-assembly of CNT using magnetically capturing residual iron (Fe) catalyst as shown in Figure 6 [71]. The Fe catalyst provides a seeding site on one end of the CNT after the synthesis. Using a Ni pattern on the electrode, this Fe catalyst is magnetically attracted to the edge of Ni pattern. The assembled CNT is finally aligned parallel to the flow direction by fluidic shear force. This simple technique enables exploring the pristine characteristics of CNT without any pretreatment for assembly of the sensor. Also, power connection or complex equipment is not necessary to fabricate the device. As a result, this technique can be applied to fabricate a highly sensitive biosensor using specifically functionalized CNT.

The microfabricated biochip combined with nanomaterials can achieve high sensitivity with all the advantages of LOC such as high throughput, low cost fabrication, and fast and automatic processing. In this sense, there are large demands for LOC biochips incorporating nanomaterials as a sensing component. Thus, this lab-on-a-chip device is very promising as a point-of-care device and for use at the laboratory level. Also, it is necessary for these chips to have high repeatability so the clinician can reliably work with the output from the device.

## 5. Biosensors in Cell Biology

Cell signaling networks sense changes in the microenvironment, transfer the information to intracellular components, and activate specific genes. With recent advances in understanding cell signaling networks, it is of primary importance to measure the activity and localization of the molecules involved and to define their overall function in a spatiotemporal manner [74-85]. All the cascades of biochemical reactions in the cell that are triggered by extracellular factors are controlled by second messengers that ultimately lead to the transcription of genes, protein expression, and effector functions. This cascade of events can be very rapid or it may require days to be completed, and multiple events are dynamically interconnected in a complex network. The ability of a cell to recognize and then respond to external stimuli is the foundation for normal tissue development and function, and it presides over essential processes such as wound healing and immunity. If this delicate balance is compromised, normal cells deviate into an abnormal state and diseases such as cancer, autoimmunity and osteoporosis develop. Thus, understanding the network of cell signaling in a spatiotemporal manner allows the discovery of effective disease treatments. However the problem is that proper nano and micro scale biosensors that can detect defects in signaling in a cell are not currently available on the market. A major challenge of sensors in general is to preserve the viability of the cell during the intracellular measurements.

The analysis of the localization and activity of a single protein can be currently achieved with well-established techniques. The detection of specific protein localization is currently dependent on fluorescence microscopic techniques such as a surface-enhanced Raman scattering, confocal, two photon microscopy, and total internal reflection florescence microscopy. Real-time measurement of the activity of proteins like ion channels can be obtained by electrophysiological techniques such as patch clamping [77]. Intracellular calcium, pH, and membrane potential can be measured using specific dyes or ion selective electrodes. Further modification of these approaches for use on a chip will provide opportunities for new medical devices [78-86].

To measure hundreds of proteins simultaneously is a more challenging endeavor. But this is critical to understand the function of a cell since the strength and duration of multiple protein expression and activity is important to determine the cell's fate. With the advance in nanotechnology, the development of high throughput sensor systems is an emerging area which allows quantitative data acquisition for complex systems. Metabolic sensor platforms incorporating a customized bioreactor, which allows sensing simultaneously intracellular and extracellular environment compartments, will be an important area in the future. This metabolic chip will be highly effective in drug screening and new therapy development as it will allow simultaneous monitoring of drug levels and the intracellular signaling events they trigger. The metabolic chip could also be developed in, for example, a sensing device to measure differentiation processes from a stem cell to a specific cell type.

The experimental data acquired from this physiological chip can be used to develop a dynamic model to understand the underlying mechanism of cell signaling networks. Using this model, simulation with variable parameters not possible in real experiments can provide new information and further the discovery of novel signaling pathways in cells. Matlab with a systems biology toolbox and multi-physics commercial software such as CFD ACE+, and COMSOL simulation software will be useful to develop simulation models for spatial-temporal dynamics.

Another application for these high throughput sensors is in gene analysis such as DNA/RNA chips and protein microarrays which feature thousands of microscopic spots of known DNA/RNA/protein and where target sequences are detected by fluorophore-, silver-, or chemiluminescence labeling methods. This gene chip can be incorporated within a microfluidic channel or in a mass spectroscopy system. The data generated from this chip would be used for the development of systems biology models.

### 5.1. Biosensors in Orthopedic Biology

The need for biosensors in orthopedic areas is important for addressing health related problems associated with soft tissues including tendon, ligament, and hard tissue that is bone. Bone-related diseases such as osteoporosis, Paget's disease, and renal osteodystrophy mainly result from the imbalance of the bone re-modeling process. Two types of bone cells are involved in bone remodeling; one is the osteoclast cell that removes the mineralized bone matrix and the other is the osteoblast cell that forms bone matrix following bone resorption. Thus, a cycle of bone remodeling consists of three consecutive phases: pre-existing bone resorption by osteoclasts, a reversal phase that is characterized by mononuclear cells on the bone surface, and new bone formation by osteoblasts to fill in the cavities after resorption. This turnover metabolic activity of bone remodeling can be indirectly assessed by detecting bone markers. The bone turnover markers can also be used to estimate fracture risk and to determine the response of bone to treatment of bone disease [87-91].

Biochemical markers reflecting bone remodeling include bone formation markers, for example, alkaline phosphatase and resorption markers that are associated with collagen cross-links. Among several bone turnover markers reported as listed in Table 2, Type I collagen accounts for more than 90% of the organic matrix of bone [88, 89]. During the remodeling process of bone matrix, type I collagen is degraded into small peptide fragments that are eventually secreted into blood stream. In addition, pyridinium cross-link, cross-linked *N*-terminal teleopeptides of type I collagen, and *C*-terminal teleopeptides of type I collagen are released into urine [90]. A specific amino acid sequence found in *C*-terminal teleopeptides of type I collagen has antibodies that are commercially available.

**Figure 7.**
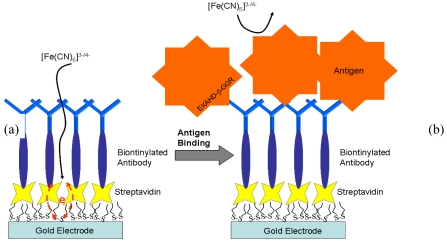
Schematic representation of a label-free immunosensor for bone turnover maker detection. Part (a) shows a self-assembled monolayer of dithiodipropionic acid deposited on a gold surface with streptavidin immobilized next as a self-assembled monolayer. Then the biotinylated antibody was bound to the streptavidin. Part (b) illustrates the antigen-antibody binding event and how it hinders the interfacial electron transfer reaction of [Fe(CN)_6_]^3−/4−^.

Figure 7 is a schematic representation of a label-free immunosensor for bone turnover marker detection. The sensor is based on a gold electrode but can be scaled down to use a carbon nantoube electrode.

**Figure 8.**
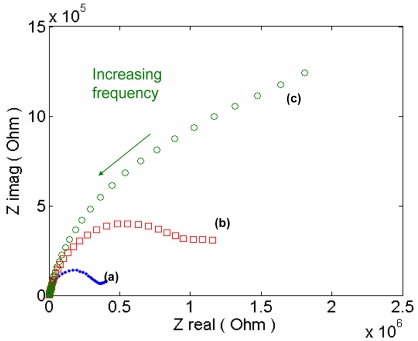
Electrochemical impedance spectra response recorded at: (a) the gold electrode, (b) the biotinylated anti-human C-terminal telopeptide antibody modified electrode, (c) C-terminal telopeptide immobilization. EIS was done at a DC potential of 0.2 V at frequencies between 0.1 Hz and 300 KHz. The sinusoidal potential magnitude was 20 mV in 5.0 mM K_3_Fe(CN)_6_ and 5.0 mM of K_4_Fe(CN)_6_ in PBS (pH 7.0).

Based on a previous electronic biosensor [91], and the design in Figure 7, a label-free electrochemical impedance spectroscopy (EIS) immunosensor for detecting bone-related degradation products of *C*-terminal teleopeptides from Type-1 collagen was developed. The response of the sensor is shown in Figure 8. In order to carry out a comprehensive study of patients to identify the metabolism of bone turnover, it would be useful to have an electrochemical biosensor as a point-of-care device that can detect several bone turnover markers at a relatively low cost. Clinical trials for multiple bone markers that can be simultaneously monitored by nano-biosensors would help to understand the significance of changes in the bone turnover markers over time and to establish a realistic measurement profile.

### 5.2. Biosensors in Cancer Biology

The early diagnosis of cancer is the most critical factor for patient survival and the treatment of cancer. Rapid detection with an ultra-low detection limit of cancer markers is important for early diagnosis of cancer. Specific protein markers for prostate, lung, breast, and colon cancers are known and listed in Table 3. DNA markers of genetic abnormalities such as germline RB, p53, BRCA I & II, APC and MMR genes are important for early diagnosis of cancers as well [92-95].

The detection of cancer markers can be done using a number of techniques-including standard immunoassays using samples from blood, urine and biopsy [96-102]. Based on the sensor's data along with correlated results from other techniques such as MRI (which tells the tumor status such as the location, grade, and stage of the tumor), the tumor can be properly treated. Detection of multiple markers using array type sensors, which can quantitatively describe the status of cancer, and lab-on chip sensors allows point-of-care service as a non-invasive technique.

With the advance of new therapies and drugs, solid benign-primary tumors can be removed using a number of methods such as surgery, chemotherapy, and radiation therapy. Surgery for complete removal of cancer tissue is often successful for small localized-cancer. However, a benign tumor can recruit endothelial cells to undergo angiogenesis and these results in multiple malignant tumors. Some cancer cells leave a primary site, disrupt the basement membrane of endothelial cells, and enter the systemic circulatory system. Once tumor cells circulate through the whole body through the blood stream, they can settle in other sites, adhere, and penetrate the basement membrane structure. Further migration of cancer cells to underlying tissues results in secondary tumor development. Thus, not only the detection of specific proteins, but also the capture of the specific cancer cells in blood circulation is very important for the early diagnosis of cancer since the invasion and metastasis of tumors are the main reasons for patient mortality. Sunitha Nagrath *et al.* [96] reported that a microfluidic chip can capture circulating cancer cells in the range of 5–1,000 cells per mL. A preliminary study to detect cancer cells using carbon nanotube electrodes in a fluidic channel is given in [97,98].

Metastasis is a complex process in a microenvironment in which cancer cells escape from the primary tumor site, circulate in the blood stream and are finally seeded at other locations. Joyce *et al.* [100] suggested the mechanism of cancer metastasis in a microenviroment including possible related cytokines, growth factors and metastases and further cancer cell invasion routes such as adhesion and penetration.

**Figure 9.**
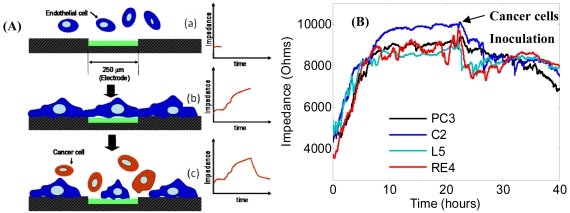
ECIS for cancer invasion study; (A) Schematic representation of an invasion assay and (B) invasion of different prostate cancer cells. Prostate endothelial cells were first incubated for 24 hours and cancer cells were added next. Impedances dramatically decreased with different slopes, which characterizes the invasion ability of each cancer cell. PC-3 is a human prostate cancer cell line which is androgen receptor negative. C2 (TRAMP-C2) is a cell line established from TRAMP tumor. RE3's full name is TRAMP-C2RE3, which is a cell line derived from C2 by recycling three times in the prostate of mice. L5 is derived from RE3, recycled five times by injection into the prostate and collecting lymph node metastasis.

The invasion properties of different cancer cell-types was studied using an electrode cell impedance sensor (ECIS). The schematic representation for the ECIS is shown in Figure 9(A) which mimics the microenvironment of cancer metastases. Endothelial cells are seeded and proliferated on the electrode and create a cell monolayer which increases the electrical impedance of the biosensor. Then this monolayer is attacked with highly metastatic prostate cell lines such as PC3, which retract endothelial cells and finally penetrate the monolayer. Figure 9(B) shows the results of the invasion properties of different prostate cancer cells. Prostate endothelial cells were inoculated first and spread on the electrode for 24 hours which increased the impedance to around 9,000 Ohms. Then different cancer cells were added and the invasion of cancer cells caused the impedance to decrease to 8,000 Ohms. The impedance after inoculating RE4 and L5 prostate cancer cells immediately decreased, showing these cells are the most invasive.

### 5.3. Implantable Biosensors

There have been a number of trials to develop implantable biosensors to detect specific bio-molecules or chemicals in the body over a period of time [103-108]. However, gradual degradation of sensitivity over time remains a challenging problem, which is caused by non-specific binding, platelet deposition and activation, wound-healing, and inflammation behavior reacting to a foreign material (the sensor). These limitations can be separated into two categories depending on where a sensor is implanted: in tissue, or in the blood stream.

**Figure 10.**
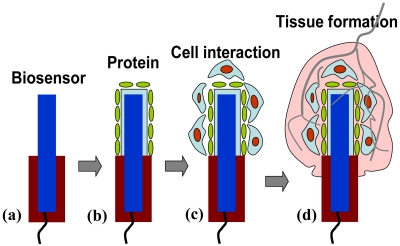
Bio-fouling progresses on the sensor's surface after implantation into the body; (a) biosensor, (b) protein absorption, (c) cell deposition, and (d) fibrosis and angiogenesis.

Biosensors which are implanted in tissue or an organ in the body should be designed considering tissue biocompatibility and inflammation behavior as depicted in Figure 10. The first response after inserting a sensor is protein absorption (such as proteins, peptides, proteoglycans, and phospholipids) which is caused by an acute inflammatory reaction. This “biofouling” behavior contributes to the loss of sensitivity and degrades the low-detection limit. Thus a sensor should be calibrated at each time by alternative methods. Especially albumin, immunoglobulin, fibrinogen, and hemoglobin peptide tightly bind to the sensor's surface and make the surface friendly for the attack of phagocytoic cells such as neutrophils, monocytes, and macrophage which try to destroy an implanted sensor. Further cell migration, adhesion through integrin receptors, and proliferation on the sensor's surface eventually creates an encapsulation by neighboring tissue such as collagen fiber or bone mineralization. For example, if a sensor is subcutaneously inserted in a mouse, fibrous encapsulation will be dominant. Angiogenesis around an inserted sensor to provide oxygen to encapsulated tissue is another important step for the wounded healing process. Biostability of an implanted biosensor such as membrane swelling, softening, fragmenting, and mineralization should be considered when the sensor is designed.

Blood biocompatibility of intravascular biosensors is referred to as the ability to detect a specific molecule for a desired time period without creating any local or systemic toxicity. Blood roughly consists of 55% plasma and 45% cells. Blood plasma is mainly water and proteins. Important soluble proteins for blood biocompatibility are albumin, fibrinogen, thrombin, immunoglobulin, fibronectin, laminin, vitronectin, and collagen (tropocollagen) which are rapidly absorbed on the sensor's surface. Platelets adhere and become activated on this absorbed layer in arterial flow. The activation of adhered platelets brings more platelets and generates coagulation, which ultimately forms blood clotting called thrombosis. Fibrinogen and fibrin in venous flow also forms thrombosis. Transport phenomenon such as diffusion is limited by thrombosis which masks proper measurement of the analyte. Furthermore, consumption of oxygen and glucose and carbon dioxide generated by metabolic activities of the attached cells interfere with the detection of a specific molecule [101-108].

Biofouling phenomenon of an implanted biosensor can be addressed by the proper; (1) choice of sensing methods and sensor miniaturization, (2) design of the sensor's shape and size, (3) choice of the sensor materials, (4) modification and coating on a sensor's surface such as an anti-inflammation drug, and (5) consideration of the time-line needed for measurements. As a non-fouling coating, glyme was used and fluoropolymer is known for low platelet reactivity. As anti-clotting agents, heparin and nitric oxide are well known as inhibitors of platelet activation and adhesion. Further use of cell-growth inhibitors such as Taxsol or non-steroidal anti-inflammation drugs is another option.

## 6. Future Biosensors

**Figure 11.**
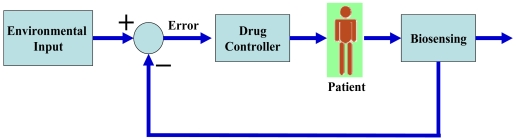
Schematic diagram of feedback controlled personalized medicine.

With the development of diagnostic technology, medical treatment can be customized to patients based on the individual's specific medical characteristics, including age, gender, height, weight, diet, and environment [109-113]. Information technology (IT) should provide a data-base for the patient's family history and life environment. This will minimize adverse effects of drugs and maximize drug efficiency [110-113]. The real success of this personalized medicine depends on whether diagnostic techniques such as biosensors can be used to obtain reliable and repeatable results in a timely manner. Point of care devices should be modified and improved for this concept. For example, bio-sensors can be wearable, implantable, provide real-time monitoring, communicate with drug injection instrumentation, and measure a drug release rate as illustrated in Figure 11. Also, adaptable biosensors can scale their sensitivity range based on the first measurement. Another challenging topic for biosensing will be the non-specific binding problem. Sensors should either simultaneously measure non-specific binding and target proteins or actively remove the non-specific binding proteins by applying external-energy.

## 7. Conclusions

Biological science in medicine is rapidly developing and now a large amount of new information is available. Great advances have been attained in the last decade in the area of intracellular signaling transduction which regulates intracellular activities and results in the secretion of cytokines and growth factors for tissue organization. Errors in a specific intracellular pathway can develop into a certain disease. The hidden roles of certain proteins and genes that cause errors in intracellualar pathways and hence diseases keep being discovering. If a sensor can detect a specific protein called a biomarker or signaling molecule in either the inside or outside of cells, early diagnosis of diseases is possible. Therefore, the role of sensors in medicine and individual medicine is becoming increasingly essential.

## Figures and Tables

**Table 1.** Phage recovery during screening Tomlinson (I and J) single chain antibody fragments (scFv) phage display libraries.

**Round Input phages (pfu)**			**Eluted phages (pfu)**		**Phage recovery**	

	**I**	**J**	**I**	**J**	**I**	**J**

1	1 × 10^12^	1 × 10^12^	1.1 × 10^6^	4 × 10^6^	1.1 × 10^−6^	4 × 10^−6^
2	3 × 10^12^	2 × 10^12^	5.5 × 10^6^	3.4 × 10^8^	1.83 × 10^−6^	1.7 × 10^−4^
3	6 × 10^12^	3 × 10^12^	4 × 10^7^	4 × 10^9^	0.67 × 10^−5^	1.33 × 10^−3^
4	3 × 10^12^	1 × 10^12^	3.5 × 10^8^	5 × 10^9^	1.17 × 10^−4^	5 × 10^−3^

**Table 2.** List of bone turnover markers that might be used in biosensors.

**Markers for bone resorption**	**Markers of bone formation**

Cross-linked telopeptides (NTx, CTx)	Total alkaline phosphatase
Pyidinolines(Pyridinoline, deoxypyridinoline)	Bone alkaline phosphatase
Hydroxyproline	Osteocalcin
Deoxypridinoline	Procollagen type I propeptides
Cathepsin K	
Tartrate-resistant acid phosphatase	

**Table 3.** Known biomarkers associated with cancer diagnosis and prognosis [101].

**Cancer type disease**	**Biomarker**
Prostate	PSA, PAP
Breast	CA15-3, CA125, CA27.29, CEABRCA1, BRCA2, MUC-1, CEA, NY-BR-1, ING-1
Leukaemia	Chromosomal abnormalities
Testicular	α-Fetoprotein (AFP), β-human chorionic gonadatropin, CAGE-1, ESO-1
Ovarian	CA125, AFP, hCG, p53, CEA
Any solid tumour	Circulating tumour cells in biological fluids, expression of targeted growth factor receptors
Colon and pancreatic	CEA, CA19-9, CA24-2, p53
Lung	NY-ESO-1, CEA, CA19-9, SCC, CYFRA21-1, NSE
Melanoma	Tyrosinase, NY-ESO-1
Liver	AFP, CEA
Gastric carcinoma	CA72-4, CEA, CA19-9
Esophagus carcinoma	SCC
Trophoblastic	SCC, hCG
Bladder	BAT, FDP, NMP22, HA-Hase, BLCA-4, CYFRA 21-1
